# Best practices for variant calling in clinical sequencing

**DOI:** 10.1186/s13073-020-00791-w

**Published:** 2020-10-26

**Authors:** Daniel C. Koboldt

**Affiliations:** 1 Steve and Cindy Rasmussen Institute for Genomic Medicine at Nationwide Children’s Hospital, Columbus, OH USA; 2 Department of Pediatrics, The Ohio State University, Columbus, OH USA

**Keywords:** Next-generation sequencing, Variant calling, Mutation detection, Clinical sequencing, Cancer sequencing, Best practices

## Abstract

Next-generation sequencing technologies have enabled a dramatic expansion of clinical genetic testing both for inherited conditions and diseases such as cancer. Accurate variant calling in NGS data is a critical step upon which virtually all downstream analysis and interpretation processes rely. Just as NGS technologies have evolved considerably over the past 10 years, so too have the software tools and approaches for detecting sequence variants in clinical samples. In this review, I discuss the current best practices for variant calling in clinical sequencing studies, with a particular emphasis on trio sequencing for inherited disorders and somatic mutation detection in cancer patients. I describe the relative strengths and weaknesses of panel, exome, and whole-genome sequencing for variant detection. Recommended tools and strategies for calling variants of different classes are also provided, along with guidance on variant review, validation, and benchmarking to ensure optimal performance. Although NGS technologies are continually evolving, and new capabilities (such as long-read single-molecule sequencing) are emerging, the “best practice” principles in this review should be relevant to clinical variant calling in the long term.

## Background

The emergence of next-generation sequencing more than a decade ago represented a major technological advance over traditional sequencing methods. NGS technologies enabled ambitious large-scale genomic sequencing efforts that have transformed our understanding of human health and disease, such The Cancer Genome Atlas [1–8], the Centers for Mendelian Genomics [9], and the UK10K Project [10]. They have also been widely adopted for clinical genetic testing. Whole-exome sequencing, which selectively targets the protein-coding regions of known genes, has become a frontline diagnostic tool for inherited disorders [11–14]. Targeted panels which leverage this approach to interrogate medically relevant subsets of genes have become core components of precision oncology [15–17].

The characteristics and sheer volume of NGS reads necessitated the development of a new generation of computational algorithms and analysis pipelines equipped to handle such data. As NGS technologies have matured, so too have the software tools for key analytical tasks, such as variant calling. Ten years and thousands of samples later, we now have a much deeper understanding of the capabilities and limitations of NGS for detecting and characterizing sequence variation. In this review, I discuss the current “best practices” for variant calling in clinical sequencing for both germline analysis in family trios and somatic analysis of tumor-normal pairs. This includes recommendations for the choice of sequencing strategy, NGS read alignment/preprocessing, combination of multiple variant calling tools, and rigorous filtering to remove false positives. I also include guidance on benchmarking NGS analysis pipeline performance using “gold standard” reference datasets to achieve the optimum balance of sensitivity and specificity.

### Sequencing strategies and implications

The choice of sequencing strategy for a clinical sample has important ramifications for variant calling (Table 1). Single- or multi-gene panels are increasingly cost-effective means of testing for subsets of genes associated with specific clinical phenotypes. For example, the OtoSCOPE hearing loss panel [18] targets 89 genes and microRNAs associated with hearing loss (1574 total exons); across a cohort of 711 sequenced patients, the average sequence depth achieved was 716× per patient. Numerous gene panels are commercially available, ranging in size from a single gene to hundreds of genes. Exome sequencing, which targets virtually all ~ 20,000 protein-coding genes, typically achieves > 100× average depth across the target regions. Whole-genome sequencing offers the most comprehensive approach and typically yields ~ 30–60× average sequence depth across the entire genome. Other considerations, such as cost and turnaround time, also influence the choice of sequencing strategy but are beyond the scope of this review.

**Table 1:** Sequencing strategies for NGS and empirical variant detection sensitivity. The Otoscope hearing loss panel v5 [18], which targets 89 genes and microRNAs, illustrates a typical gene panel. The approximate size of the total target space is given in megabase pairs (Mbp). Typical exome kits target ~ 50 Mbp of genome bases comprising coding sequences, splice sites, alternative exons, and some non-coding RNAs, though this space varies among manufacturers

Strategy	Panel	Exome	Genome
Size of target space (Mbp)	~ 0.5	~ 50	~ 3200
Average read depth	500–100×	100–150×	~ 30–60×
Relative cost	$	$$	$$$
SNV/indel detection	++	++	++
CNV detection	+	+	++
SV detection	–	–	+
Low VAF	++	+	+

Dollar signs represent approximate relative costs, though it should be noted that the cost of panel sequencing depends on the size of the panel. The empirical performance of each strategy for detecting variants of different classes is indicated as good (+), outstanding (++), or poor/absent (−)

These differences in depth and breadth of sequencing coverage have implications on variant calling. All three strategies generally offer excellent sensitivity for detecting SNVs/indels using tools such as GATK HaplotypeCaller [19] and Platypus [20]. Copy number variants (CNVs) spanning multiple exons can be called with reasonable sensitivity using panel and exome data [21]. Whole-genome sequencing remains the superior strategy for the comprehensive detection of all types of sequence variants. However, it should be noted that the higher sequence depth achieved in panel and exome sequencing may enable more sensitive detection of variants at low allele frequencies, e.g., subclonal somatic mutations in cancer and mosaic germline variants [22–24].

### Alignment and pre-processing

The primary analysis of sequencing data, including its alignment to a reference sequence, is a critical phase of NGS analysis. A selection of recommended tools can be found in the top of Table 2.

**Table 2:** Key components of NGS analysis and a list of exemplar tools. Most clinical sequencing pipelines will employ a single read aligner (e.g., BWA-MEM) and mark duplicates with one algorithm (e.g., Picard). However, multiple tools for collecting sequencing metrics and performing sample QC may be employed to meet the needs of the laboratory. For variant calling, it is recommended that pipelines incorporate 2–3 tools for each class of variant to maximize detection sensitivity. See the relevant section of this review for recommendations specific to each variant class

Strategy	Variant callers
**Alignment and pre-processing**	
Read alignment	BWA-MEM [25], Bowtie 2 [26], minimap2 [27], Novoalign
Marking duplicates	Picard tools [28], Sambamba [29], SAMBLASTER [30]
BAM file creation	Samtools [31], GATK [19]
Sequencing metrics	BEDTools [32], Picard tools [28], QualiMap 2 [33]
Sample quality control	KING [34], VerifyBamID [35]
**Variant calling**	
Inherited SNVs/indels	FreeBayes [36], GATK HaplotypeCaller [19], Platypus [20], Samtools/BCFtools [37]
Somatic mutations	deepSNV [38], MuSE [39], MuTect2 [40], SomaticSniper [41], Strelka2 [42], VarDict [43], VarScan2 [44]
Copy number variants	cn.MOPS [45], CONTRA [46], CoNVEX [47], ExomeCNV [48], ExomeDepth [49], XHMM [50]
Structural variants	DELLY [51], Lumpy [52], Manta [53], Pindel [54], SVMerge [55]
Gene fusions (RNA-seq)	fusionCatcher [56], fusionMap [57], mapSplice [58], SOAPfuse [59], STAR-Fusion [60], TopHat-Fusion [61]
**Variant review/storage**	
Visualization and review	Artemis [62], Integrative Genomics Viewer [63]
VCF/BCF file manipulation	BCFtools [37]

*BAM* binary alignment/map, *SNV* single nucleotide variant, *VCF* variant call format, *BCF* binary variant call format

**Fig. 1:**
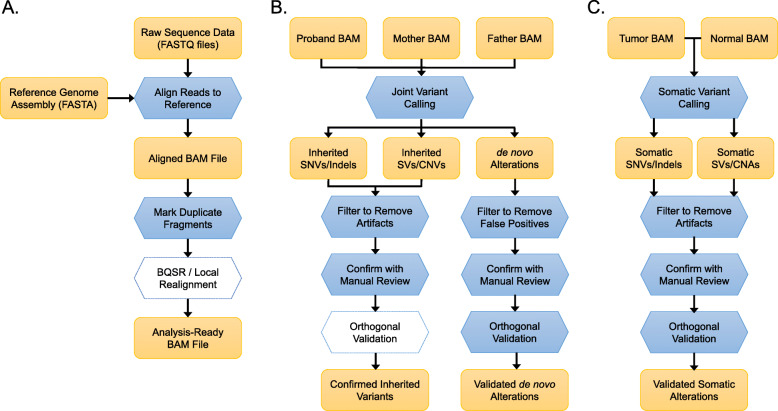
Standard pipelines for NGS analysis. **a** Alignment and pre-processing of NGS data for an individual sample. Raw sequence data in FASTQ format are aligned to the reference sequence, with the resulting alignments typically stored in binary alignment/map (BAM) file format. Marking of duplicates in the BAM file is a critical step to account for duplicate reads of the same fragment. Base quality score recalibration (BQSR) and local realignment around indels are a computationally expensive step that may marginally improve variant calls. At the conclusion of this step, the file is ready for variant analysis. **b** Variant calling in NGS trio sequencing. In this common study design, variants are called jointly (simultaneously) in a proband and both parents, which enables the phasing of variants by parent of origin. The initial variant calls are typically filtered to remove a number of recurrent artifacts associated with short-read alignment and maybe visually confirmed by manual review of the sequence alignments. Orthogonal validation may be performed to confirm the variant and its segregation within the family. De novo alterations should be aggressively filtered to remove both artefactual calls in the proband (false positives) and inherited variants that were under-called in a parent (false negatives). In addition to manual inspection of alignments, most de novo mutations are independently verified by orthogonal validation techniques, such as Sanger sequencing. **c** Somatic variant calling in matched tumor-normal pairs. Identification of somatic alterations in tumors requires specialized variant callers which consider aligned data from the tumor and normal simultaneously. Candidate somatic variants are filtered and visually reviewed to remove common alignment artifacts as well as germline variants under-called in the normal sample. The resulting variants are typically validated by orthogonal approaches, which may require specialized approaches for low-frequency variants

In a typical pipeline (Fig. 1a), raw sequence data in FASTQ format are aligned to the reference sequence using an aligner such as BWA-Mem [25], with the resulting alignments typically stored in binary alignment/map (BAM) file format [31]. Because of their compressed file size, indexed-access capabilities, and standardized data formats, BAM files have become the standard format for storing and sharing NGS data. The Samtools package [31] provides most of the BAM file manipulation tools required for clinical sequencing.

Once NGS data are aligned to the reference sequence, it is possible to identify redundant reads that originated from the same DNA sequence molecule. These “PCR duplicates” represent 5–15% of sequencing reads in a typical exome [64] and can be identified on the basis of the alignment position and read pairing information. Tools such as Picard [28] and Sambamba [29] identify and mark duplicate reads in a BAM file to exclude them from downstream analysis.

The GATK Best Practices workflow [65] recommends two additional steps for pre-processing BAM files prior to variant calling. The first is base quality score recalibration (BQSR), which adjusts the base quality scores of sequencing reads using an empirical error model. The second is local realignment around indels, which aims to reduce false-positive variant calls caused by alignment artifacts (discussed below). Evaluations of variant calling accuracy before and after BQSR/realignment suggest that the improvements are marginal [66]; because of this and the high computational cost, this may be viewed as an optional step for pre-processing.

Routine quality control (QC) of analysis-ready BAMs should be performed prior to variant calling to evaluate key sequencing metrics [28], to verify that sufficient sequencing coverage was achieved [32], and to check samples for evidence of contamination [35]. In the case of family studies and paired samples (e.g., tumor-normal), expected sample relationships should be confirmed with tools for relationship inference such as the KING algorithm [34].

### Benchmarking resources for variant calling

Evaluating the accuracy of variant calls requires access to benchmark datasets in which the true variants are already known. Several such benchmarking resources have been made publicly available in recent years. The most widely used ones include the Genome in a Bottle (GIAB) [67] and the Platinum Genome [68] datasets for NA12878, a human sample of European ancestry that has been sequenced with various technologies at laboratories around the world. Each benchmarking dataset includes a set of “ground truth” small variant calls (SNVs and indels) based on the consensus of several variant calling tools, as well as defining the “high-confidence” regions of the human genomes in which variant calls can be benchmarked against a variety of public resources. The GIAB dataset has been continually improved with the addition of data from multiple short-read and linked-read sequencing datasets and the expansion of the reference from one sample to seven [69]. The Global Alliance for Genomics and Health has also established a best practice framework to guide evaluations of variant calling accuracy using these resources [70]. As discussed in this paper, sophisticated comparison tools which account for subtle differences in variant representation are recommended when comparing a set of variant calls against a benchmark resource.

One drawback of the aforementioned benchmarking resources is that many of the same sequencing technologies and variant calling algorithms evaluated against them were also used to construct the reference datasets in the first place. Synthetically created datasets in which the positions of all sequence variants are known a priori have been published to address this issue. For example, the synthetic diploid (Syndip) dataset is derived from de novo long-read assemblies of two homozygous human cell lines and aims to provide a less biased view of variant calling accuracy genome-wide [71]. Syndip is uniquely advantaged to provide benchmarking data for more challenging regions of the genome, such as duplicated sequences. Although the cell lines themselves are not in a public repository, sequencing datasets for both are widely available. More guidance on using benchmarking datasets to optimize variant calling performance is offered in the relevant sections below.

## Best practices for germline variant calling

Dozens of variant calling tools for NGS data have been published in the past 10 years, and countless more have been developed by researchers for internal use. A selection of exemplar tools grouped by purpose can be found in the middle of Table 2. Because SNV/indel detection tools such as GATK HaplotypeCaller have demonstrated high accuracy (*F*-scores > 0.99) in numerous benchmark datasets, choosing a single variant caller that meets the needs of the laboratory (in terms of pipeline compatibility and ease of implementation) is usually sufficient. However, combining the results of two orthogonal SNV/indel callers, such as HaplotypeCaller and Platypus, may offer a slight sensitivity advantage. Software packages such as BCFtools make it possible to merge and reconcile multiple variant callsets (in VCF format) into one, though care should be taken to properly handle complex variants and/or differences in variant representation [70].

To discuss the recommended best practices for germline variant calling, we will consider trio sequencing for inherited disorders, which is a common scenario for clinical genetic testing. A trio analysis pipeline typically begins with the analysis-ready BAM files for the proband and both parents (Fig. 1b). For optimal results, all three samples should be sequenced under identical protocols (capture kit, instrument, and reagent kit) and processed with identical alignment and pre-processing steps. This is particularly important for copy number variant calling and SV calling, which rely on uniform sequencing depth and library insert size, respectively.

### Individual versus joint variant calling

Virtually, all variant calling tools can be applied to individual samples after alignment and pre-processing are complete. It may be preferable, therefore, to perform variant calling on every sample as it comes through the pipeline. Doing so can facilitate automation of NGS analysis, which may be desirable for laboratories processing large numbers of samples. Individual VCF files can be merged later using BCFtools or similar packages; however, it should be noted that VCF files typically only contain entries for positions that are variant in a particular sample. In other words, when a variant is only detected in some samples but not others, it is not clear whether the other samples are wild type for that position or simply did not achieve sufficient coverage for the variant caller to make a call.

Joint variant calling—which considers all samples simultaneously—offers several key advantages. First, it produces called genotypes for every sample at all variant positions, not just the ones that were detected in a given individual. This makes it possible to differentiate between a position that matches the reference sequence with high probability and a position in which the sample did not achieve sufficient coverage. Second, in the case of trio sequencing, joint calling enables direct inference of phase information to establish, for example, whether two heterozygous variants in a proband are in *cis* or in *trans*. Third, it mitigates the issue of variant representation differences which might otherwise be problematic, particularly for complex variants [72]. Finally, joint analysis allows a variant caller to use information from one sample to infer the most likely genotype in another, which has been shown to increase the sensitivity of variant calling in low-coverage regions [19].

### SNV/indel calling

Numerous tools have been developed to identify single nucleotide variants (SNVs) and short insertions/deletions (indels) from aligned NGS data. Most tools for this purpose, such as Samtools/BCFtools [37] and FreeBayes [36], employ Bayesian statistics to infer the most likely genotype. GATK HaplotypeCaller [19] and Platypus [20] also employ local realignment or assembly of sequencing reads to improve the accuracy of variant calls. Numerous studies have compared the relative performance of these tools on various datasets and have found, generally, that they produce similar results: variant concordance is typically 80–90% concordance or higher, with most differences are attributed to variants at low-coverage or low-confidence positions [73–76]. Even so, such differences could amount to thousands of variant calls genome-wide. Thus, it is important not only to choose a robust variant caller for SNVs/indels, but also to benchmark and fine-tune it to achieve optimal performance on the data to be analyzed.

### Filtering to remove artifacts

The accuracy of NGS variant calls relative to the previous “gold standard” of Sanger sequencing has been well documented at > 99% [77–79]. However, it should be noted that NGS data are prone to certain types of artifactual variant calls, many of which are related to errors in short-read alignment [37, 66]. Numerous groups including ours have investigated the source of artifacts and demonstrated that they can be systematically filtered without significantly compromising sensitivity [41, 44]. Even so, visual review of the alignments for clinically relevant variants, using a tool like the Integrative Genomics Viewer [63], is recommended to identify false-positive variant calls that slip past automated filters.

**Fig. 2:**
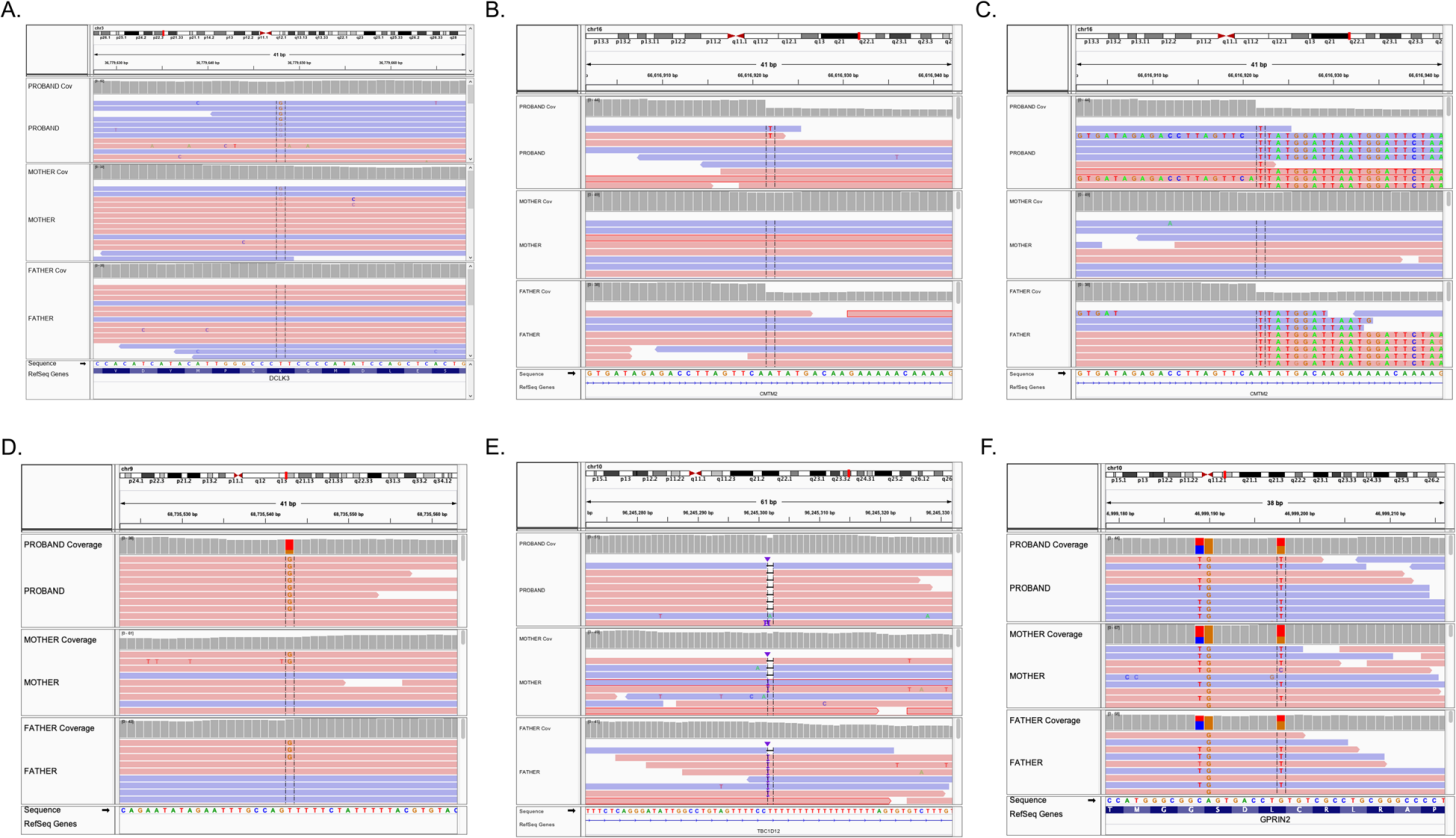
Common artifacts in NGS alignments that gave rise to a false-positive de novo mutation call in a family trio. Each pane is an IGV screenshot of WGS alignments for the proband (top track), mother, (middle track), and father (bottom track). Each sample’s track comprises two parts: a histogram of the read depth and the reads as aligned to the reference sequence. Reads are colored according to the aligned strand (red = forward strand; blue = reverse strand). **a** False positive associated with low base quality. Most reads supporting the variant have low base quality indicated by lightly shaded non-reference bases. Four reads in the proband showed the alternate allele with good quality, triggering the variant call. **b** False positive due to misalignments near the start or end of reads. Notice that the alternate allele is only observed at the start/end of reads in the proband. In this case, the read depth histogram provides a clue as to the cause of the misalignment. As shown in the next panel, this occurs at the breakpoint of a large paternally inherited deletion. **c** The same position as in **b**, but with soft-clipped bases shown in color. BLAT alignment of such reads reveals that the soft-clipped portion matches the other side of the deletion segment some 5.2 kb downstream. **d** False positive associated with strand bias. All but one variant-supporting reads in the proband are on the reverse strand, whereas reference-supporting reads are equally represented on both strands. **e** False positives associated with low-complexity sequences. In this case, reads erroneously showing a single-base deletion (horizontal black line) at a T-homopolymer are enriched in the proband. R supporting insertions (purple) are also seen. Note that this position is zoomed out compared to the other panels, a recommended practice to visualize the end of repetitive sequences. **f** False positives due to paralogous alignments of reads from regions not well represented in the reference. Alignments for proband include reads with several substitutions relative to the reference sequence within the 41-bp viewing window. This typically occurs when reads from sequences not represented in the reference are mapped to the closest paralog

Figure 2 depicts several frequently occurring artifacts that can be identified by manual review: low-quality base calls (Fig. 2a), read-end artifacts (Fig. 2b) due to local misalignment near indels (Fig. 2c), strand bias artifacts (Fig. 2d), erroneous alignments in low-complexity regions (Fig. 2e), and paralogous alignments of reads not well represented in the reference (Fig. 2f).

### Orthogonal validation of NGS variants

Whether or not Sanger confirmation should be required for clinically relevant variants remains a matter of debate [80, 81]. In general, the validation rate for NGS variant calls is extremely high—99.965% according to a well-powered study [79]—suggesting that for the vast majority of NGS variants, independent confirmation is unnecessarily redundant. In many cases, a visual manual review of the variant may be enough to determine if it passes muster or warrants orthogonal validation. An interlaboratory study of more than 80,000 clinical specimens demonstrated that a heuristic approach examining fewer than ten criteria (read depth, quality score, observed variant allele sequence, repetitive sequence, etc.) can identify the subset of variants most likely to be false positives and thus requiring orthogonal validation [82].

### Identifying de novo mutations

A key advantage of joint calling in trios is the ability to distinguish de novo mutations, which account for a significant proportion of positive diagnoses from clinical genetic testing [11, 83–85]. According to recent large-scale trio sequencing studies, the human de novo mutation rate is approximately 1.29 × 10^−8^ per base pair per generation [86, 87]. Thus, each proband likely harbors ~ 70 de novo mutations genome-wide against a background of ~ 4–5 million inherited variants. In the protein-coding exome, we expect ~ 1 de novo mutation on a background of ~ 50,000 inherited variants. A sequence variant called in the proband is therefore far more likely to be inherited than de novo. Furthermore, even with extremely high variant calling precision (99.9%), there will be 50 false-positive calls for each de novo mutation. Thus, candidate de novo mutations merit careful scrutiny.

In addition to filtering for artifactual calls as described above, de novo mutations should be queried against public databases of genome variation, such as the gnomAD database. Although true de novo mutations can certainly occur at positions of known sequence variants, a candidate de novo with appreciable frequency in the population (i.e., MAF > 0.0001) is far more likely to represent a germline variant. Similarly, manual review in Integrative Genomics Viewer (IGV) should be used to exclude both artifactual calls and variants with supporting evidence in one or both parents (e.g., Fig. 2a).

### Copy number and structural variant calling

Copy number variants (CNVs) are a major source of human genetic variation and have been implicated in numerous diseases [88–90], such as autism [91], intellectual disability [92], and congenital heart disease [93–95]. Although microarray testing is typically ordered prior to panel or exome testing in a clinical setting, NGS-based CNV detection is increasingly incorporated into clinical diagnostic testing and accounts for 3–5% of positive diagnoses. A number of tools exist for identifying CNVs from targeted NGS data, such as cn.MOPS [45], CONTRA [46], CoNVEX [47], ExomeCNV [48], ExomeDepth [49], and XHMM [50]. Most rely on comparisons of sequence depth between a test subject and a comparator to identify significant changes in copy number. Not all CNV calling tools perform well in all situations, and as a rule, the sensitivity for CNV detection using targeted NGS is limited compared to genome sequencing [96].

Paired-end whole-genome sequencing data also enables the detection of structural variants with increasing precision. Popular tools for this application, such as DELLY [51], Lumpy [52], Manta [53], Pindel [54], and SVMerge [55], use two types of information to identify signatures of structural variants. Read pairing information serves to identify segments of the genome in which molecularly linked read pairs map at unexpected distances or orientations. Split read alignments, in which a single sequence read maps to two different regions of the genome, are also incorporated into SV calling. It should be emphasized that while many consider SNV/indel detection with NGS to be routine, SV detection with whole-genome sequencing data is still challenging, as illustrated by the fact that leading tools achieve F-1 values of only ~ 0.80–0.90 in benchmarking experiments. There are at least two principal reasons for this. First, it is widely recognized that a large proportion of structural variation occurs in “difficult” regions of the genome, such as repetitive or tandem-duplicated sequences. Second, the relatively short length of NGS reads (~ 150 bp) and typical fragments (~ 300–500 bp) is often insufficient to resolve complex structural variants and long insertions [97]. For this reason, linked-read and long-read sequencing technologies are increasingly being applied to resolve large SVs and complex sequences [98–100], for a recent review, see [101].

**Fig. 3:**
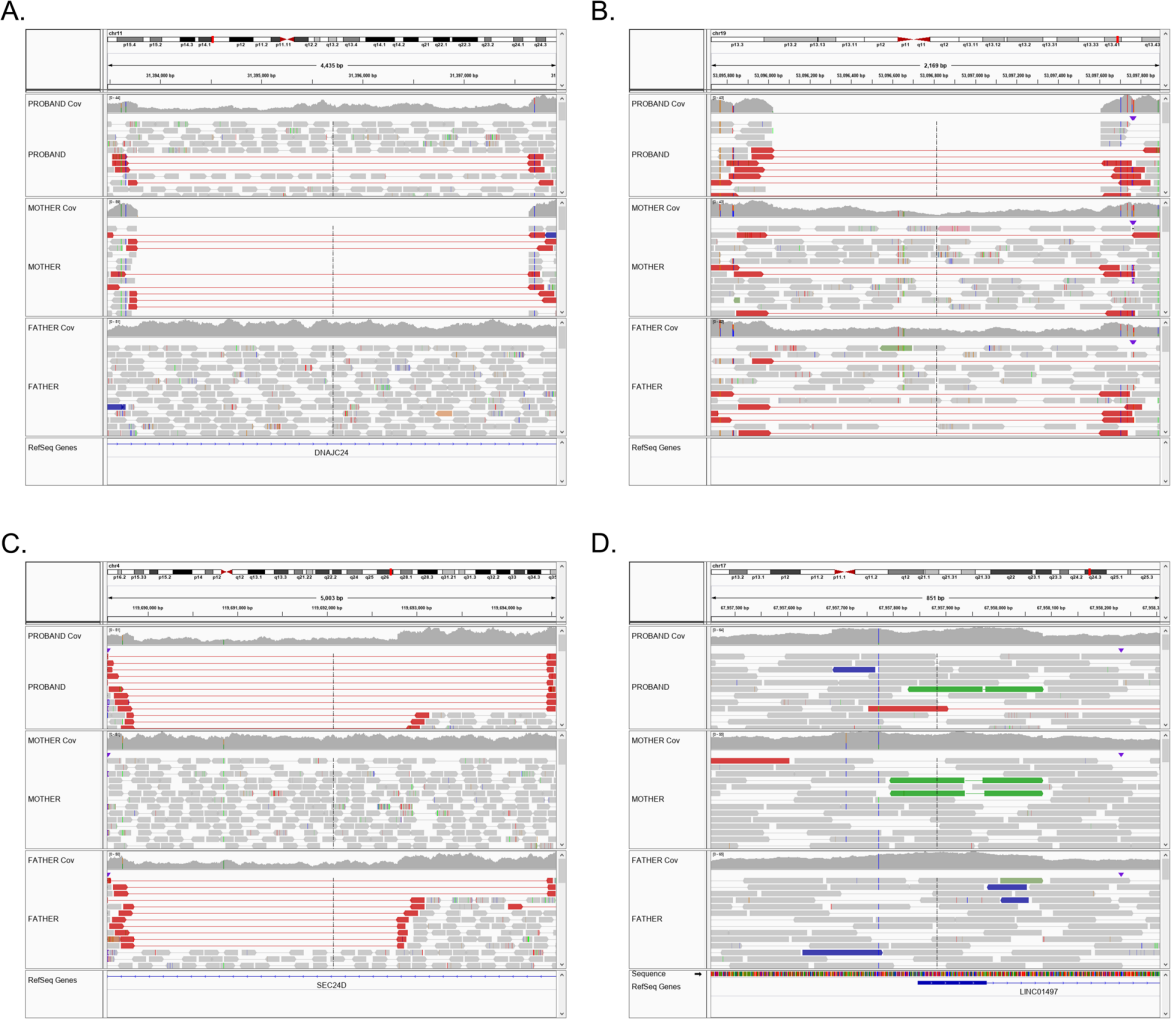
Visual review of copy number and structural variants. Each pane is an IGV screenshot of WGS for a proband (top), mother, (middle), and father (bottom). The top track for each sample is a histogram of sequence depth. Reads are viewed as pairs, with discordant pair alignments highlighted in color. **a** A homozygous ~ 4-kb del that appears heterozygous in the proband, homozygous in the mother, and absent from the father. Note the discordant read pairs suggesting a deletion (red) and visible change in read depth. **b** Homozygous deletion inherited from two heterozygous parents. **c** A heterozygous paternally inherited deletion with ambiguous end point by paired-end mapping resolved by visual inspection of read depth. **d** A maternally inherited tandem duplication. Note the increased read depth in the histogram and the discordant read pairs highlighted in green that span the original sequence and their tandem duplication

Visual review of CNVs and structural variants called by NGS can also, to some extent, be performed in IGV. For SVs in particular, it is useful to view reads as pairs and color them according to insert size, as shown in Fig. 3. Well-supported structural variants are often supported by both discordant read pairs and changes in overall sequence depth, such as the deletions in Fig. 3a and b and the duplication in Fig. 3d. Manual review can also help resolve ambiguous SV breakpoints (Fig. 3c).

### Benchmarking germline variant calling pipelines

As described in the previous section, several reference datasets and a “best practice” framework for benchmarking variant calling pipelines are publicly available. At the time of writing, the most recent dataset for sample NA12878 includes ~ 3.04m SNVs and ~ 0.5m small indels, as well as aligned high-depth Illumina sequencing data in BAM format. These resources make it possible to evaluate performance and fine-tune variant calling pipelines to achieve optimal results. For small variants, an F1 score > 0.99 should be achievable by robust variant calling pipelines. High-quality DNA samples for NA12878 can also be ordered from Coriell and sequenced independently to evaluate the performance of a laboratory’s entire pipeline from sample preparation through variant calling.

Benchmarking structural and copy number variant callers tends to be more challenging for two reasons. First, these variants are more challenging to detect with precision using short-read sequencing data. Second, the precise breakpoints for SVs/CNVs are not always well-defined, which makes comparisons across callsets a more complex endeavor. Even so, multiple “gold standard” SV callsets such as GIAB [99], HS1011 [102], and HuRef [103] have been published which employ orthogonal sequencing technologies to define reference callsets comprising thousands of structural variants. When benchmarking with such resources, it is important to recognize that SV calling with short-read data is more error-prone than small variant calling; even the best-performing SV callers only achieve F-1 scores of ~ 0.80–0.90 [103].

## Best practices for somatic mutation calling

NGS of tumor specimens is increasingly deployed in oncology to guide diagnosis, prognosis, and personalized care [104]. Although ~ 10% of cancer patients harbor germline predisposition variants, the main purpose of clinical tumor sequencing is often the identification of somatic mutations, copy number alterations, and fusions that may have clinical relevance. A standard pipeline for this is shown in Fig. 1c. It illustrates a paired tumor-normal sequencing strategy, that is, sequencing DNA from a tumor sample and a matched control sample (e.g., blood or skin) from the same patient. Although tumor-only sequencing has been adopted by many laboratories as a cost-effective approach to guide cancer diagnosis, prognosis, and therapy [16, 105–107], doing so makes it difficult to distinguish true somatic mutations from constitutional variants [108–110]. Thus, the emphasis of this section will be on the “best practice” of sequencing a tumor sample with a matched comparator sample.

Numerous variant callers have been published for this purpose; a list of the most cited callers can be found in Table 2. Widely used somatic mutation callers, such as MuTect2 [40], Strelka2 [42], and VarScan2 [44], consider aligned data from the tumor and normal simultaneously. Several groups have attempted to directly compare the performance of mutation callers for different applications [111–113], finding that each has strengths and weaknesses. Because no somatic caller has emerged which offers superior performance in all scenarios, an ensemble approach that combines the results of two or more complementary callers may offer the best balance of sensitivity and specificity [73, 114].

Several aspects of clinical tumor sequencing can make the detection of somatic mutations more challenging. Tumor purity—the proportion of cells in a sample that are cancerous—governs the representation of somatic mutations in a sequenced sample, but pathology estimates of purity based on light microscopy are notoriously inaccurate [115–117]. Somatic mutations present at low frequency due to low tumor cellularity and/or subclonal mutation architectures can be challenging to detect, even with high-depth sequencing data. Although many somatic mutation callers such as VarScan2 can be configured for the detection of variants at low frequencies, doing so often reduces the overall false-positive rate. The type of specimen obtained for sequencing also influences mutation calling. Formalin-fixed, paraffin-embedded (FFPE) samples, which are preferred for histopathological diagnosis, often harbor thousands of artifacts arising from chemical DNA damage [118–120]. These challenges call for a robust somatic mutation detection pipeline that performs well across many types of clinical tumor samples.

### Filtering somatic variant calls

Similar to germline SNVs/indels, candidate somatic variants should be filtered to remove common alignment artifacts such as those illustrated in Fig. 2. In addition, the availability of a matched normal sample enables a direct comparison of data characteristics at the site of a candidate somatic variant call to help distinguish true variants from false positives. For example, reads supporting high-quality mutation calls should exhibit similar position and strandedness as reads supporting the wild-type allele. Other metrics, such as the difference in average mapping quality or trimmed read length, help uncover false positives due to alignment artifacts. Mismatch quality sum (MMQS) difference, computed as the average sum of base qualities for non-reference base calls in variant-supporting reads, is a powerful metric for identifying false positives associated with paralogous alignments [121].

### Filtering with population databases

Population variant filtering is a powerful strategy for identifying and removing likely germline variants from somatic mutation callsets but should be done with caution. Simply removing all variants in dbSNP [122] is an appealing but hazardous strategy, since that database contains a number of recurrent mutations from human tumors—such as p.(H1047R) in *PIK3CA* (rs121913279) and p.(R132H) in *IDH1* (rs121913500)—as well as several mutations from the COSMIC somatic mutation database [109]. There is a similar risk for applying a broad filter based on all variants in the gnomAD database [123], in which the presence of apparent somatic loss-of-function variants in hematological malignancy genes like *ASXL1* has been documented [124]. Allele frequency information can be used to safeguard against the inadvertent filtering of true somatic variants that are present in such databases. Requiring a minimum minor allele frequency > 0.0001 in the gnomAD or TopMed database is recommended to select variants for filtering somatic mutation callsets.

Some groups have also found value in using an internal “panel of normals” to identify and remove recurrent sequencing artifacts [38]. In this approach, sequencing data from a set of normal DNA specimens (typically ~ 50) are compiled into a reference panel against which candidate somatic variants from tumors can be quickly filtered to remove variant calls associated with germline variants or sequencing artifacts. This approach is advantageous because it identifies artifacts that may be specific to a laboratory’s sequencing protocols or downstream analysis pipelines.

### High-confidence somatic SNV/indel calls

In summary, high-confidence somatic SNV/indel calls should be identified by multiple somatic mutation calling tools at positions with sufficient sequencing coverage (> 10× in both tumor and normal tissue). Variant alleles should be supported by reads on both strands with no apparent bias in read position, base quality, or mapping quality. High-quality SNVs/indels should also be absent from public databases and an internal laboratory panel of normal (if available), or else present at very low frequencies (MAF < 0.001). Finally, candidate SNV/indel calls should be reviewed by visualization of the tumor and normal sequencing alignments with a tool such as IGV.

### Calling somatic copy number and structural variants

**Fig. 4:**
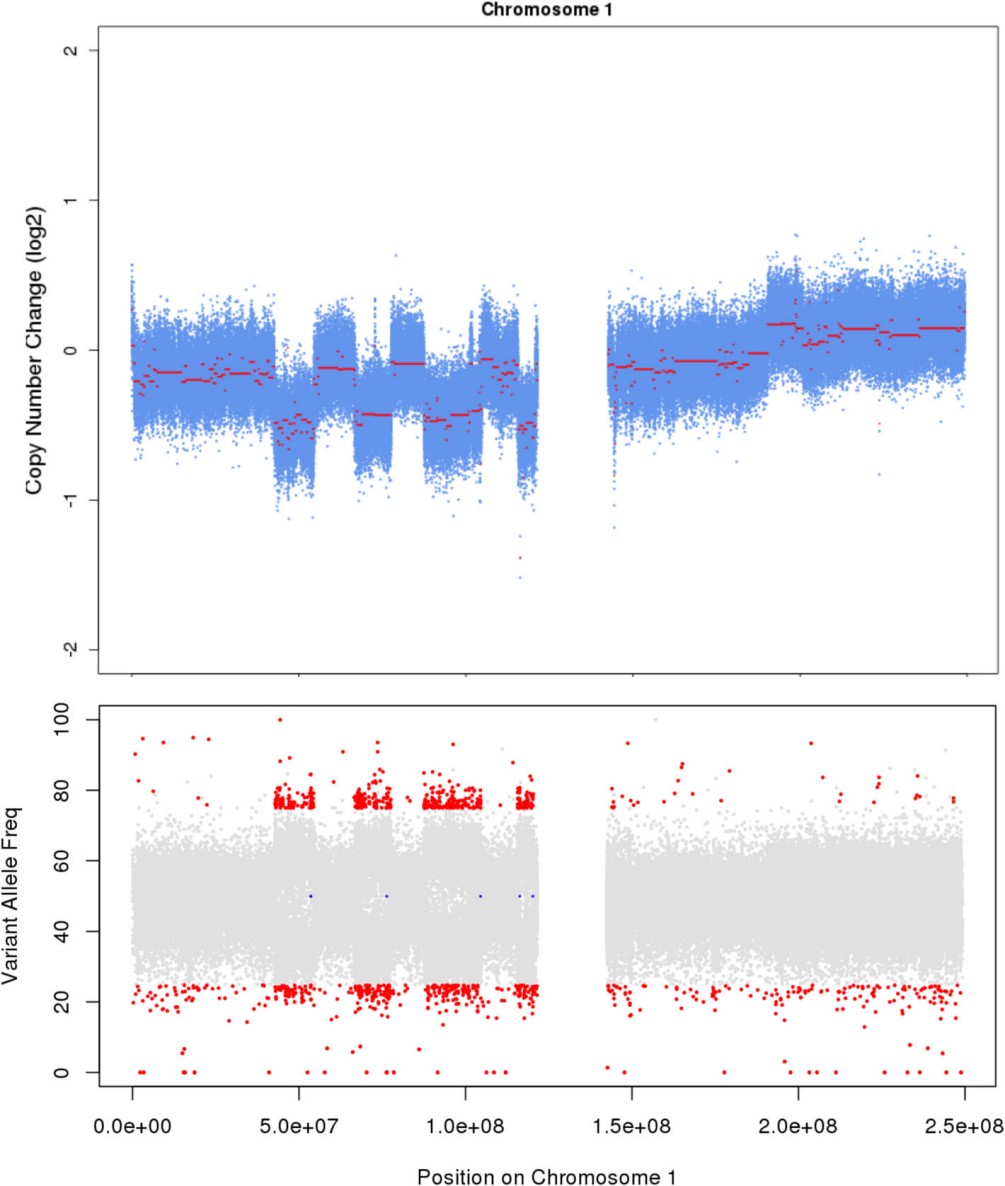
Detecting somatic rearrangements in cancer using NGS. Shown is whole-genome sequencing data for chromosome 1 for a tumor-normal pair. Top: Log2 values indicate copy number changes in the tumor relative to the normal. Bottom: copy gains and losses skew tumor allele frequencies for heterozygous variants, with loss of heterozygosity (red) apparent in regions of heterozygous deletions

Many of the tools developed for germline CNV/SV calling have been adapted for cancer genomics [125], and still, others have been developed for the critical task of identifying fusions from RNA-seq data [126]. Somatic copy number alteration (SCNA) detection is arguably the easier of the two tasks, since a matched normal sample is often alive to use as a comparator. Further, deep sequencing data allow for precise determination of variant allele frequencies, the skewing of which can often be observed to support candidate variants. Similar to somatic mutation calling, combining the results of at least two tools, such as VarScan 2 (less conservative) and GATK (more conservative), may provide the optimal strategy for calling somatic CNAs. Further, incorporation of tumor variant allele frequency (VAF) information can help generate supporting evidence for somatic structural variants, since changes in copy number tend to skew allele frequencies of heterozygous variants (Fig. 4). Similar to somatic SNV/indel calling, somatic SV/CNA calls may be filtered against a panel of normals to remove calls in regions of highly variable copy number and recurrent artifactual SVs.

### Benchmarking somatic calling pipelines

Benchmarking somatic mutation callers requires a reference “truth set” of real somatic mutations. Such datasets have been generated by synthetic mixing experiments (for example, of NA12878 with another well-characterized sample at specifically defined proportions). Of note, though numerous comparisons of somatic mutation callers have been published, the findings are inconsistent [127]. One reason for this is that the researchers conducting those studies often apply variant callers with default parameter settings or neglect to perform critical downstream filtering. To address this issue, the DREAM ICGC-TCGA Somatic Mutation calling challenge invited teams, including several developers of somatic mutation calling tools, to benchmark their pipelines on a common dataset. The organizers employed a robust simulation framework to introduce synthetic somatic alterations (i.e., a truth set) into real WGS data for three tumors upon which each team’s submissions were evaluated. The simulated datasets and truth sets from these challenges are freely available and offer a well-vetted benchmarking resource for somatic SNV, indel, and structural variant calling pipelines [128].

## Conclusions and future directions

Variant calling in NGS data, much like NGS technologies themselves, has evolved considerably over the past decade and remains an active area of research. Robust pipelines for NGS analysis include steps for optimized alignment and pre-processing, variant calling, filtering of false positives, and visual manual review. While some of these procedures, such as read alignment and SNV/indel detection, can be suitably performed with a single software package, others, such as CNV/SV calling and somatic mutation detection, benefit from incorporating multiple independent tools. Benchmarking resources for both germline and somatic variants provide an opportunity to evaluate and optimize the performance of variant calling. Although some classes of variants—such as de novo mutations in germline studies and low-frequency somatic mutations in cancer patients—likely require validation on an orthogonal platform, the burden of additional confirmatory testing is likely to decrease as technologies continue to improve. However, the observation that even state-of-the-art SV callers only achieve F-scores of ~ 0.80–0.90 in gold standard datasets suggests that emerging long-read sequencing technologies may ultimately be required to accurately call large and/or complex structural variants. Nevertheless, the general principles discussed in this review—rigorous pre-processing of sequencing data, implementation of multiple variant calling approaches, and systematic filtering to remove artifacts—will remain relevant guidance for clinical variant calling in years to come.

## Data Availability

Not applicable

## Abbreviations

BAM: Binary alignment/map
CNV: Copy number variant
NGS: Next-generation sequencing
SNV: Single nucleotide variant
VCF: Variant call format
